# An Introduction to the Study of Diseases of the Nervous System

**Published:** 1885-10

**Authors:** 


An Introduction to the Study of Diseases of the Nervous System, *being Lectures delivered in the University of Edinburg during the Ter-centenary Year. By* Thomas Grainger Stewart, m. d., f. r. c. p. e., f. r. s. e., *etc. Philadelphia: J. B. Lippincott & Co. Chicago: W. T. Keener.*

We have in this short and practical guide to the study of nervous diseases a very valuable contribution to the literature of the subject. There are not wanting large and valuable treatises on nervous affections, but until the appearance of the present work there has been none in our language that would serve as a fitting introduction to their study. The first three lectures are devoted to the medical anatomy of the nervous system; the next three to sensory functions; the seventh and eighth to motor funtions, and one each on electricity in diagnosis, vaso-motor, secretory and trophic functions, cerebral and mental functions, language, pathology and general treatment. The lecture on speech and its disorders is, for brevity and clearness, the best we have ever seen.

The book is well written, typographically excellent, and freely illustrated. We note with pleasure the reproduction of Charcot's illustration of an acute bed-sore and the joint-changes in ataxia. As they have been in use fully twenty years and appear in our three most recent works (Bromwell, Ross and the present one), they bid fair to outlive most of Charcot's other works. H. N. M.

